# “Whoooo said that?”: responses of captive owls (*Strigiformes*) to the voices of familiar caregivers

**DOI:** 10.7717/peerj.21421

**Published:** 2026-06-17

**Authors:** Gina Montalto, Jennifer Vonk, Victoria L. O’Connor, Lauren Velazquez, Cameron Ferguson

**Affiliations:** 1 Department of Psychology, Oakland University, Rochester, Michigan, United States; 2 Bergen County Zoo, Paramus, New Jersey, United States

**Keywords:** Owl, Vocal discrimination, Heterospecific recognition, Caregiver relationship

## Abstract

Group-living is hypothesized to select for individual recognition, but territorial and cooperatively breeding species would also benefit from this ability, even with regard to heterospecifics. Some social species differentiate familiar human caregiver voices from the voices of unfamiliar humans but few non-domesticated, non-group-living species have been tested. We presented 21 captive owls (*strigiformes*) representing seven species with playbacks of unfamiliar and familiar human voices. We predicted that subjects would respond (*i.e*., head turns, peering, vocalizations and postural changes) differently to familiar compared to unfamiliar voices but that this response would depend on the nature of the relationship between the owls and their caregivers. The owls were slower to respond and increasingly likely to exhibit fearful postures across playback trials within sessions. However, owls were more likely but slower to respond to the voices of familiar caregivers the longer they had worked with them for. They were also faster to respond to the voices of those that performed aspects of their husbandry and those that rated their relationships with the owls more positively. Lastly, they were less likely to show fear when they heard the voices of their trainers compared to those of other familiar individuals and when the relationship was rated as more positive compared to less positive. These results suggest that owls in managed care are capable of recognizing individual caregiver voices, and even that they may encode aspects of their relationship along with the sound of the caregiver’s voice.

## Introduction

Recognition of familiar individuals is beneficial for group-living animals to reduce conflict and competition, coordinate territorial defense, forage collectively, and engage in alloparenting. Although it might be expected that individual recognition should be selected for in larger, more complex groups in which more complex communicative signals might evolve (Peckre, Kappeler & Fichtel, 2019), tests of less social species must be carried out to confirm whether such abilities are specific to group-living species. It is less clear whether members of non-group-living species would benefit from the ability to discriminate familiar from unfamiliar individuals. However, individual recognition may aid territorial species in responding appropriately to neighbors *vs*. strangers (Lambrechts & Dhondt, 1995; Temeles, 1994), and may facilitate mate recognition in cooperative breeders (Lee, McIvor & Thornton, 2025; Rosell & Bjørkøyli, 2002). Other hypotheses that focus on factors such as sibling competition in the emergence of individually distinctive calls (Dreiss, Ruppli & Roulin, 2014) further emphasize the importance of studying non-group-living species. In addition, recognition of heterospecifics is less well understood compared to recognition of conspecifics even though animals that routinely interact with heterospecifics likely benefit from the ability to differentiate non-threatening familiar individuals from potentially threatening strangers (Marzluff et al., 2010). This may be especially true for animals in managed care that depend upon humans for survival.

Currently, it is unknown how widely distributed the capacity to differentiate human voices is within the animal kingdom, and the extent to which an animal’s relationship with humans may influence this capacity. In species bred to work closely with humans, such as domestic dogs (*Canis familiaris*), responsiveness to human communicative cues, including recognition of familiar human voices, may be selected for through the process of domestication (Hare, Call & Tomasello, 1998; Hare & Tomasello, 1999, 2005). Discrimination of familiar human voices has been demonstrated in various domesticated species; *e.g*., dogs (Root-Gutteridge et al., 2019; Surányi et al., 2025), cats (*Felis catus*, Saito & Shinozuka, 2013; de Mouzon, Gonthier & Leboucher, 2022), and goats (*Capra aegagrus hircus*, Baciadonna et al., 2019). However, the ability has also been demonstrated in related but non-domesticated species; Grey wolves (*Canis lupus*, Gammino et al., 2023), cheetahs (*Acinonyx jubatus*, Leroux et al., 2018) and various other cat species (*Felidae*, Crews, Vonk & McGuire, 2024), suggesting that the ability is not limited to domesticated species or those that are naturally group-living. Most species tested to date have shown stronger responses to familiar humans compared to unfamiliar humans, perhaps because the sound of a caregiver’s voice is associated with food or enrichment and therefore elicits interest. For example, cats and wolves responded more quickly and intensely to playbacks from familiar compared to unfamiliar human voices (*e.g*., Crews, Vonk & McGuire, 2024; Gammino et al., 2023; Leroux et al., 2018; Saito & Shinozuka, 2013). However, two bird species—ringed Australian magpies (*Gymnorhina tibicen dorsalis*, Dutour et al., 2021) and captive carrion crows (*Corvus corone corone*, Wascher et al., 2012)—showed increased response, which the researchers referred to as vigilance (*i.e*., head and body movement to the speaker, looking up and changing into a vigilant position) to the unfamiliar voices. These different patterns may reflect the extent to which humans are primarily associated with provision of food or exposure to threat, which may be a function of species differences or individual experiences. Ontogenetic experiences may be more critical in shaping this ability compared to domestication.

Whereas carnivores in managed care have responded more strongly to familiar *vs*. unfamiliar human voices, for animals that perceive humans as threats, habituation to a familiar voice may lead to a reduction in stress and a reduced response, rather than a faster or more intense response as described above. Animals rescued from the wild may come to interact with humans because of injuries sustained by humans or human artifacts, such as vehicles and nets. Raptors frequently come into conflict with humans and may be generally wary, but may come to exhibit reduced response to familiar humans that provide food, care, or engage in extensive interactions with them. Owls often come to live under managed care because they have been injured and rehabilitated by humans but are deemed non-releasable (Trumpp et al., 2024). We observed owls in managed care, some of whom engaged in extensive training with human caregivers, to explore the role of the caregiver/owl relationship in determining their response to familiar caregiver voices. Owls that are imprinted to humans early in life are less likely to display signs of distress around humans and are generally easier to train (Potts, 2016) suggesting that they may show reduced responses to familiar human voices.

In addition to their varied history with humans, owls are of interest because they are a diverse group with varied habitats, physiology, social structures and relatively unknown cognitive abilities. Acoustic communication is critical for many nocturnal species (Madhavan et al., 2025; Mikkola & Mikkola, 2022) and several owl species are known to produce a number of diverse call types (*e.g*., barred owls (*Strix varia*), Odom & Mennill, 2010a), making them good candidates for studies of vocal recognition (Madhavan & Linhart, 2025). Owls possess a heightened ability to recognize the source of a sound—even when the source is concealed. They possess specialized visual and auditory structures that permit brain mapping of auditory space (Knudsen & Konishi, 1978) and assist in the precise capture of prey (Payne, 1971). Indeed, hearing is the primary modality used to detect prey for these nocturnal hunters (Trumpp et al., 2024). Given the relatively limited dependence on olfaction in birds and the low light conditions at night, vocal communication is especially critical for nocturnal birds (Madhavan & Linhart, 2025). Low-amplitude vocalizations may be the primary form of communication in the forested environment that owls often occupy (Peixoto, de Paiva & Gonzaga, 2025).

The facial ruff exhibited in many owl species consists of a disk of feathers acting as a parabolic reflector to collect sound energy and enhance sensitivity (Lynch, 2007). The minimum auditory sensitivity of most owls is roughly 25 decibels lower than that of other bird species (Lynch, 2007) with a minimum sensitivity for American and European barn owls (*Tyto alba guttata*) of −14.2 dB. Lower values reflect greater sensitivity. Both species also exhibit a higher upper-frequency limit than other bird species with audiometric studies showing the greatest sensitivities between 4 and 8 kHz, whereas they can respond to frequencies from 200 up to 13 kHz (Dyson, Klump & Gauger, 1998; Kraemer et al., 2017). It has been suggested that the typical owl is most sensitive to frequencies between 2–4 kHz, with Eastern screech owls most sensitive to frequencies between 1.5 and 6.4 kHz (Brittan-Powell et al., 2005). The adult female human voice typically falls within 150–200 Hz, which is outside of the peak sensitivity but clearly detectable by owls, making them appropriate candidates for the current study.

The fact that many species of owls form pair bonds makes them good candidates for individual vocal recognition. Generally, owls are not considered to be social until the breeding season occurs. Many owl species like the Eurasian eagle owl (*Bubo bubo*) are socially monogamous and will continue to pair bond with same mate in each breeding season (AZA Raptor Taxon Advisory Group, 2022). However, whether owls bond with a different mate in each breeding season varies across species and could depend on migratory patterns and prey availability (Colbourne, 2019). Polygamy has been reported in the snowy owl (*Bubo scandiacus*), northern hawk owl (*Surnia ulula*), and boreal owl (*Aegolius funereus*, Johnsgard, 1989). Pair bonding seems critical for successful rearing of the young of some species. Interestingly, barn owls that lose a mate have been known to take on a new mate during the rearing process with the new mate assisting with parenthood (AZA Raptor Taxon Advisory Group, 2022). Female owls appear more likely to incubate than their mates, but further observation is required to validate this claim (Johnsgard, 1989). In some owl species, the male is known to assist with the rearing of their offspring by providing the female with food while she incubates, which she then feeds to her offspring (Johnsgard, 1989). In snowy owls, it is common for the male to hunt for food and deliver the food to the female while she remains at the nest once her young have hatched (Kaufman, 1996). Owls may exhibit features of cooperative breeding such that older owlets typically share their food with their younger siblings (Johnsgard, 1989). Cooperative breeding has been noted as a key feature shaping the evolution of cognition in primates (Burkart & Van Schaik, 2010, although see Thornton & McAuliffe, 2015), and is hypothesized to play a role in avian cognition as well (Emery et al., 2007).

Calling behaviors are an important feature of courtship and defensive communication in owls (Hardouin et al., 2007). Courtship calls, which normally occur while owls are perched near each other before copulation, may also take place during flight (Johnsgard, 1989), suggesting that owls recognize conspecific calls from long distances. Importantly, there is evidence of distinct individual calls in owls suggesting that they are capable of differentiating individuals on the basis of vocal information alone (*e.g*., in barn owls (*Tyto alba*) Dreiss, Ruppli & Roulin, 2014; great horned owls, Odom, Slaght & Gutiérrez, 2013; tawny owls, Roccazzello et al., 2024; barred owls, Tseng, Hodder & Otter, 2024; little owls (*Athene noctua*), Madhavan et al., 2025, Sunda scops-owl (*Otus lempiji*), Grieco, 2023; Yee et al., 2016; great grey owls (*Strix nebulosa*), Rognan, Szewczak & Morrison, 2009; and Eastern screech owls (*Megascopsasio*), Cavanagh & Ritchison, 1987; Nagy & Rockwell, 2012). Indeed, there is evidence of individual discrimination of mates, siblings (Dreiss, Ruppli & Roulin, 2014) and neighbors (Cavanagh & Ritchison, 1987; Grieco, 2018; Hardouin et al., 2007). Males and females may also be differentiated by their calls (Odom & Mennill, 2010a).

Odom & Mennill (2010b) examined neighbor-stranger discrimination or the dear-enemy effect in barred owls (*Strix varia*). Although barred owls exhibited increased calling and duetting rates for an extended period of time as a territorial response to the playback of duets, there were no significant differences in their response to neighbor and stranger duets. Spotted owls (*Strix occidentalis*, Waldo, 2002) also failed to differentiate between neighbors and strangers. In contrast, Galeotti & Pavan (1993) found that tawny owls (*Strix aluco*) reacted more strongly to the calls of “stranger” males compared to the calls of their familiar neighbors. Thus, there is mixed evidence for whether owls discriminate the calls of familiar from unfamiliar conspecifics. Owls in managed care likely interact more closely with caregivers than they do with neighbors in the wild, and humans may be more likely to be perceived as threats, leaving it an intriguing question as to whether they attend to the identity of individual caregivers.

Owls are commonly housed in rehabilitation centers, zoos, and nature centers, exposing them to many human voices daily and they may be designated as program or ambassador animals, which may make them more likely to differentiate individual humans. Ambassador programs involve more extensive interactions with the public as ambassador animals may be used in educational presentations, which involve training and sometimes, off-site travel. Exhibit animals typically do not participate in travel or presentations for the public, but may still experience training to perform actions that aid in husbandry routines, such as perching, or spreading their wings. Little is known about how owls perceive their relationships with caregivers. We examined whether owls in managed care could differentiate the voices of familiar caregivers from those of unfamiliar humans by analyzing their response to playbacks. Because there is no existing information on whether owls can recognize human voices, the first step is to show that they broadly differentiate between familiar and unfamiliar voices. Secondly, if owls respond differently to caregivers that engage with them to different degrees (*e.g*., cleaning enclosures *vs*. training for ambassador programs), this might constitute some preliminary evidence of individual recognition. Differences in response from ambassador and exhibit owls could be informative about how the nature of these experiences shape their perception of humans. If owls show stronger responses in response to keepers that engage in extensive training with them, this would be an important indicator of welfare and may inform decisions about involvement in ambassador programs.

Unlike carnivores, but similar to other bird species tested previously, we predicted that owls may respond less, rather than more, to familiar voices, which might indicate some degree of habituation. Given differences in investment in offspring, the fact that there are differences in song production in male and female songbirds (Henry et al., 2017), which could impact reception, and previous findings of sex differences in individual recognition (in domestic horses, *Equus caballus*, Proops & McComb, 2012) we explored the possibility of sex differences. Further, we examined responses to familiar caregivers that differed in their relationships with the owls. Some birds were program/ambassador animals that worked closely with human trainers and other birds experienced their caregivers strictly in the context of general husbandry (*i.e*., exhibit owls). We expected that owls that worked closely with caregivers in the context of training, for longer periods of time, and with more positive relationships would show reduced responses to their caregivers’ voices compared to strangers’ voices. We did not anticipate or analyze species differences based on the species in our sample, and the small number of individuals of each species.

## Method

### Ethics statement

The experiment was reviewed and approved by Oakland University’s IACUC (Protocol # 2023-1196).

### Subjects

With permission from four locations (two nature centers, a zoo and a sanctuary), we tested 21 owls of seven species (Table 1). Subjects were either ambassador (*n* = 6) or exhibit (*n* = 15) owls, with one owl housed off exhibit. Ambassador owls participated in educational programs at the nature centers, which involved being trained to perch on the trainer’s hand and take food from the trainer in the presence of members of the public. In contrast, exhibit owls never directly interacted with the public or left their home habitats. All owls were accustomed to hearing the voices of many unfamiliar humans during the day, as all owls were housed in facilities that were open to the public only during daytime hours. All enclosures housed one to two owls and contained perches, as well as naturalistic components such as tree stumps, native flora, dirt, mulch, and sand.

**Table 1:** Details about the subjects.

Common name	Scientific name	Actual name	Sex	Role	Location	Housing	Number of sessions
Barn Owl	*Tyto alba*	Gilly	Male	Ambassador	Howell Nature Center	Singly	10
Barn Owl	*Tyto alba*	Alfie	Male	Exhibit	Bergen County Zoo	Paired	4
Barn Owl	*Tyto alba*	Spanky	Female	Exhibit	Bergen County Zoo	Paired	4
Barred Owl	*Strix varia*	Sam	Female	Ambassador	Stage Nature Center	Paired	8
Barred Owl	*Strix varia*	Arguile	Male	Exhibit	Stage Nature Center	Paired	8
Barred Owl	*Strix varia*	Athena	Female	Exhibit	Howell Nature Center	Singly	10
Eastern Screech Owl	*Megascops asio*	Rito	Male	Ambassador	Stage Nature Center	Singly	8
Eastern Screech Owl	*Megascops asio*	Gizmo	Male	Exhibit	Bergen County Zoo	Singly	4
Eurasian Eagle Owl	*Bubo bubo*	Hagrid	Male	Exhibit	The Creature Conservancy	Paired	8
Eurasian Eagle Owl	*Bubo bubo*	Minerva	Female	Ambassador	The Creature Conservancy	Paired	8
Eurasian Eagle Owl	*Bubo bubo*	Winston	Male	Exhibit	The Creature Conservancy	Singly	6
Great Horned Owl	*Bubo virginianus*	Tufts	Male	Off-exhibit	Bergen County Zoo	Singly	4
Great Horned Owl	*Bubo virginianus*	Jezebel	Female	Exhibit	The Creature Conservancy	Singly	8
Great Horned Owl	*Bubo virginianus*	Autumn	Female	Ambassador	Stage Nature Center	Singly	8
Great Horned Owl	*Bubo virginianus*	Arch	Male	Ambassador	Howell Nature Center	Singly	10
Great Horned Owl	*Bubo virginianus*	Xena	Female	Exhibit	Howell Nature Center	Singly	10
Snowy Owl	*Bubo scandiacus*	Hedwig	Male	Exhibit	Bergen County Zoo	Singly	4
Snowy Owl	*Bubo scandiacus*	Citga	Female	Exhibit	Howell Nature Center	Paired	10
Snowy Owl	*Bubo scandiacus*	Yeti	Male	Exhibit	Howell Nature Center	Paired	10
Spectacled Owl	*Pulsatrix perspicillata*	Dexter	Male	Exhibit	Bergen County Zoo	Paired	4
Spectacled Owl	*Pulsatrix perspicillata*	Velma	Female	Exhibit	Bergen County Zoo	Paired	4

### Materials

Audio recordings were gathered from at least one current caregiver with whom the owls were very familiar using a Zoom H1n Handy recorder (Zoom, San Jose, CA, USA). The recording consisted of the typical greeting: “Hello, how are you doing today?,” which lasted at least 5 s. We gathered recordings of the same phrase from each familiar caregiver and from each unfamiliar individual. A total of 28 female voice samples were used, each from a different person. Nine voices were unfamiliar to all owls, whereas 19 voices were familiar to some of the owls but used as unfamiliar stimuli at other locations. We ensured that familiar keepers had not also worked with owls housed at different locations. Tone and volume were uniform and neutral for all recordings. All recordings were taken in a quiet room with no other interruptions or sounds.

A complete audio file for each session was created using the open-source software Audacity (Version 3.3; Muse Group and Contributors, 2023) to ensure consistent timing and volume between stimuli. Recordings were stored on an Apple iPad or iPhone (Apple Inc., CA, USA, 2024), which was connected wirelessly to a Beats pill (Apple Inc., CA, USA, 2024) Bluetooth speaker that was placed outside of the subject’s habitat in an area just out of sight of the subject(s). GoPro cameras (GoPro, San Mateo, CA, USA) were used to record the owls’ behavior during each trial. If necessary, multiple cameras were used to obtain a complete view of the habitat.

Familiar caregivers completed a survey to provide information about their history with the owls. We asked how long caregivers had worked with each owl in months and how often they were a primary caregiver to the owl in an average week. To assess how each familiar caregiver interacted with each owl, we asked about the role they played in working with each subject which included feeding, cage cleaning, participating in programs and training. Each of these items were collapsed into either a husbandry category (feeding, cage cleaning), or a training category (participating in programs, training) to create dichotomous variables of husbandry (no, yes) and training (no, yes). Lastly, the survey asked caregivers to rate their relationship with each subject from their own perspective using a five-point Likert scale (1 = strongly disagree to 5 = strongly agree) based on the following adjectives: comfortable, antagonistic, friendly, warm, and aloof. Aloof and antagonistic were reverse scored such that higher scores represented relationships that were perceived to be more positive from the caregivers’ perspectives.

### Procedure

Each subject or pair of subject(s) was presented with auditory playback stimuli from speakers placed outside their habitat. Each subject was presented with a minimum of two 5-playback sessions so that each familiar voice was presented for two sessions. The number of sessions presented to each subject depended upon how many caregivers’ voices were available (Table 1). For example, if there were greetings recorded from three different caregivers, we conducted six sessions. For ten of the subjects, a block of sessions included one session with each familiar voice before any familiar voices were repeated, with the sessions occurring in different random orders within blocks. For 11 subjects, the order of presentation of these sessions was not counterbalanced, due to experimenter oversight. Sessions occurred approximately 1 to 7 days apart and no more than two sessions occurred on the same day. At two locations (BCZ and TCC), testing took place in June 2023, whereas at the other two locations (HNC and SNC), testing took place between Oct. and Nov., 2023.

Each session consisted of five playbacks. The first three playbacks consisted of greetings from three different, unfamiliar individuals to avoid pseudoreplication. The fourth playback consisted of the familiar caregiver’s greeting. The final playback within a session consisted of a fourth unfamiliar person’s voice, to determine if any change in behavior across playbacks was specific to the familiar voice or reflected a temporal change throughout the session. Specific unfamiliar voices were not presented more than twice across all sessions for a given subject. This was to ensure the owls did not become habituated to specific voices over the course of the experiment. Voices were presented in different orders across sessions, with birds being presented with different random orderings of voices to disrupt any effects of the ordering of particular voices. Audio playback was of sufficient volume to be audible throughout the testing area, but not loud enough to startle any nearby animals.

Sessions were conducted during times when the owls were considered to be active by their caretakers—and few external sounds/other visitors were present. At SNC, HNC and TCC, sessions took place between 14:00 and 19:00 h whereas at BCZ, sessions took place between 08:00 and 16:00. Therefore, ambient light conditions, although consistent within a session, may have varied non-systematically between sessions. Sessions did not occur when food had just been introduced, or other major distractors were present. Familiar care staff were not present during testing although the playbacks occurred during times of day when humans are normally present so that the playbacks would not be likely to elicit surprise. On each session, the experimenter, whose voice was not included in the session, ensured that subjects were within view and began videorecording. The experimenter did not speak to or engage with the owl during the sessions and was out of the owls’ line of sight. They voiced the date, session number, and subjects’ names at the beginning of each recording. Playback began 2 min into the recorded observation with 30 s intervals between each greeting. Video recording continued for 2 min following the fifth playback. Thus, each session lasted approximately 7–10 min, and the 2 min at the beginning and end of each session served as control observation periods during which no playback occurred. Each bird at a facility received one session before any owl received a second session on a given test day. Testing took place one to two times per week over consecutive weeks such that all testing at a given facility occurred during the same season.

### Behavioral coding

All behaviors listed in Table 2 observed during each session were recorded by two naïve coders using the behavioral coding software BORIS v.8.20 (Friard & Gamba, 2016). If two owls shared a habitat, the video was coded twice; once with each animal as the focal subject. In addition to vocalizations, we coded orienting responses like head turns and movement, such as moving along a perch or flying, based on previous research. Palleroni & Hauser (2003) played prey vocalizations to adult and juvenile harpy eagles (*Harpia harpyja*), which, like owls, have specialized facial ruffs to enhance sound localization. Juvenile and adult eagles turned more to the right to attune to the acoustic cues of conspecifics, and more to the left to attune to prey vocalizations, (*e.g*., howler monkey (*Alouatta seniculus*)). We coded behaviors including head movements such as swaying and bobbing indicative of “peering,” head orientation, movement toward or away from the speakers, and vocalizations that were likely to be elicited in response to recognition of caretakers using an ethogram created for this study (Table 2). We coded the duration and frequency of these behaviors. For reliability purposes, a second coder coded a randomly determined 18.7% of the 151 videos.

**Table 2:** Behavioural ethogram used for coding.

Behavior	Code	Description of behavior	Modifiers
Playback begins	0	Audio playback begins	N/A
Head movement	1	Subject’s head moves from original position, either laterally (left to right, swaying) or vertically (up and down, bobbing)	Towards Speaker: Subject’s head turns to directly face the speaker, based on the location of the speaker. Back: Subject’s head turns to face behind their original position Left: Subject’s head turns to face the left (would be your left) Right: Subject’s head turns to face the right (would be your right) Up: Subject’s head turns to look up, towards the sky/roof of enclosure Down: Subject’s head turns to look down, towards the ground
Flapping	3	The bird is flapping its wings while remaining on its current perch.	N/A
Locomotion	4	Subject actively moves within enclosure, by flying, moving around on their perch	Moving Perch: The bird moves from one perch to another in a controlled manner; the bird may turn and look at the new perch before moving to it. Movement can be done by walking, hopping, or flying. Flying out of frame: The bird flies off of the perch out of frame, and returns to the same spot afterwards Towards Speaker: Subject moves towards the speaker Back: Subject moves away from the camera Right: Subject Moves to the right (would be your right) Left: Subject moves to the left (would be your left) Stop: Subject has stopped moving, ends the locomotion behavior
Vocalization	5	Subject makes an audible, vocalization, *e.g*., hoot, screech	N/A
Background noise	6	Audible noise coming from the habitat’s surroundings that is NOT the human voice you will hear over the speaker and does not come from the owl itself, *e.g*., other human voices, equipment like lawn mowers, car noises.	N/A
Social behaviors	7	Grooming (preening)—The bird is running its beak along and/or lightly biting its feathers or the feathers of another bird.	Self-Grooming: The bird is running its beak along and/or lightly biting its OWN feathers. Grooming Partner: The bird is running its beak along and/or lightly biting the feathers of another bird. Pecking: The bird is using its beak to strike or bite another bird. **Distance from another owl** In contact with the other bird: Less than 3″ from each other. Not in contact with the other bird: Within 6″ from each other.

Two different naïve coders later coded the same sessions for posture (0 = neutral, 1 = fearful, 2 = aggressive) as described by Johnsgard (1989). For example, coders were told that owls in a neutral posture would have unfluffed feathers, wings relaxed at the side of the body, ear tufts (if present) relaxed but not flat or hidden and that eyes could be open or closed but upper eyelids would be slightly lowered. Frightened/threatened owls were described as exhibiting the “camouflage” pose, standing upright with eyes shut or almost shut, erect ear tufts, forehead feathers spread, and body feathers compressed. Aggressive/threatening owls were described as having fluffed feathers with a spread tail and wings with wings raised above the back. The owl may be swaying, hissing or clacking its beak and its eyes would be open. Posture was coded immediately upon the onset of the playback. Coders were instructed to code any change in posture during the playbacks but there were no instances where this occurred, and the majority of trials were coded as neutral. A second coder coded a randomly determined 180 (24%) of the 750 playbacks for posture.

Cohen’s κ was conducted to determine if there was agreement between Coder 1 and Coder 2 on the outcome of the presence or absence of a behavioral response for all double-coded trials, (*n* = 134). A behavioral response could include any behaviors from the ethogram that occurred immediately following the playback, but 527 of the observed 548 responses involved head movements, such as peering. Therefore, we were unable to analyze the particular behaviors separately and decided to follow other researchers (*e.g*., Crews, Vonk & McGuire, 2024; Wascher et al., 2012) in considering any kind of head or body movement or vocalization as a response. Moderate agreement was obtained; κ = 0.61. For posture, Cohen’s κ was 0.21, indicating only fair agreement. In an attempt to resolve disagreements and improve reliability, GM recoded the sessions on which the two coders disagreed. We used GM’s coding of these trials as the secondary data and recalculated Cohen’s kappas. The kappa for posture improved to 0.51. Thus, we retained the primary coder’s data.

We recorded latency to the first observable response to the playback. A response could include head movement, locomotion, or vocalizations that occurred within the 30-s window between each playback. A Spearman’s rank-order correlation was conducted to determine the level of agreement between coders on latency to respond because the data were not normally distributed. There was a strong, significant positive correlation between coders for the videos that were double-coded, (ρ (132) = 0.690, *p* < 0.001).

### Analytic strategy

All analyses were conducted in IBM Statistical Package for the Social Sciences (SPSS) v. 30. To determine whether the owls responded differently to familiar or familiar voices, generalized linear mixed models (GLMMs) were conducted on the dichotomous variable of whether any response occurred following each playback using a binomial distribution and a logit link function. For this model, subject sex (female = 0, male = 1), status (exhibit = 0, ambassador = 1), playback (where the fourth playback was familiar and playbacks 1–3 and 5 were unfamiliar), and all two and three-way interactions were included as fixed effects. Simple contrasts were used to test whether responses to the familiar playback differed from responses to the unfamiliar playbacks. Model fit using corrected AIC was compared to models excluding any effects of sex and a null model including only the random effects (all model comparisons appear in Tables S1–S6). Parameter estimates for the best fitting models are shown in Tables S7–S12. To reduce the likelihood of overdispersion, the model specification included random effects for subject, random effects for playback nested within subject and random effects for session nested within subject to account for non-independence.

We then selected only those trials on which owls had made a response, and examined the latency to make these responses with an additional generalized linear mixed model (GLMM). We used a linear distribution with the same fixed and random effects and model structure. Homoscedasticity of the residuals for latency was examined visually by plotting residuals against fitted predicted values. Normality was assessed with q-q plots. Komolgorov-Smirnov tests confirmed that the data were not normally distributed, but GLMMs are reasonably robust to non-normality so we did not apply a transformation of the data.

We conducted a binomial GLMM of posture (neutral *vs*. fearful) on the full data set using the same effects and model structure as above.

Because individual owls may have had different patterns of response to the familiar and unfamiliar voices, and distinct responses to different familiar voices, we further examined the relationship between individual owls and the caregivers that provided the familiar voices. To determine whether certain features of the owl/caregiver relationship predicted the owls’ responses to the familiar voices, separate GLMMs were conducted on the owls’ responses, latency to respond if a response was made, and posture for only the familiar playbacks with subject as a random factor and the following fixed factors to describe the owls’ relationships with the familiar caregivers; months of time spent working with the owl (months), relationship quality as indicated by the caregiver (relationship), husbandry (no, yes), and training (no, yes).

## Results

### Do owls differentiate familiar from unfamiliar voices?

#### Response

Model fit for the full model for response was indicated by a corrected AIC of 4,388.560. There were no significant effects (Table S7). Furthermore, the simpler model without sex (AIC = 4,345.097) and the null model (AIC = 4,280.66) showed improved model fit compared to the full model (Table S1).

#### Latency to respond

**Figure 1:**
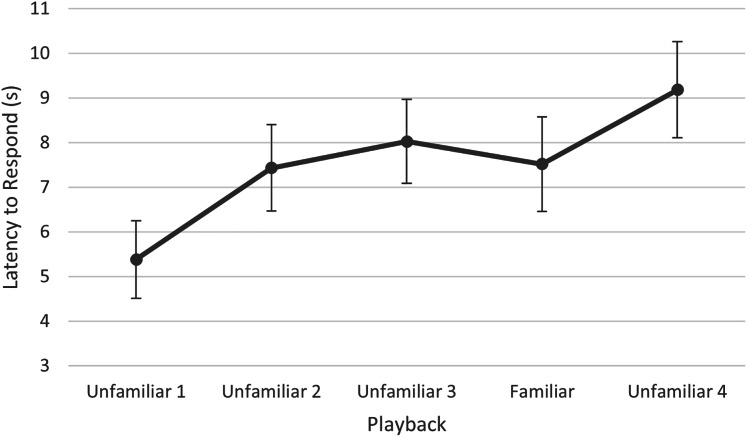
Estimated marginal means for latency to respond across playbacks when response was made. Error bars reflect standard error.

A GLMM was conducted with latency to respond for the playbacks to which a response was made as the outcome using a linear distribution with an identity link function with the same random and fixed factors as above. Model fit for the latency to respond was indicated by a corrected AIC of 3,624.400. This was a better fit than the simpler (AIC = 3,666.970) or null models (AIC = 3,707.969, Table S2) so we report the effects from the full model (Table S8). There was only a significant main effect of playback (*F*_4, 527_ = 3.911, *p* = 0.004), with owls becoming slower to respond as the playbacks continued throughout a session. Simple contrasts revealed that only latency to respond on the first playback differed significantly from that on the playback of the familiar voice (*t*_527_ = −2.044, *p* = 0.041, 95% CI [−4.187 to −0.083]). However, Fig. 1 shows that the owls habituated over the first three playbacks, responded more quickly to the familiar playback, and then responded more slowly again to the final playback.

#### Posture

**Figure 2:**
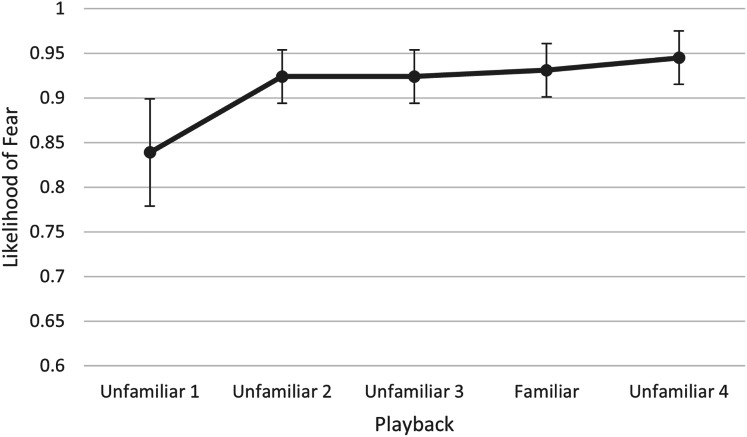
Estimated marginal means for fearful posture across playbacks. Error bars reflect standard error.

We conducted another GLMM with posture as the outcome and a binomial distribution with log link function using the same fixed factors of status, sex, playback and their interactions with subject and playback included as nested random effects. We did not include the random effect of session in this model because the model did not converge. Because there were no reliable observations of aggression, we used the dichotomous response of neutral (0) *vs*. fearful (1) as the outcome. The model fit was indicated by a corrected AIC of 4,280.212. There were no significant effects. The simpler model excluding sex had a better fit, with a corrected AIC = 3,690.604 (Table S3). In this model (Table S9), there was only a significant main effect of playback, (*F*_4, 684_ = 2.518, *p* = 0.040) with owls tending to be less fearful on the first compared to the familiar playback (*t*_684_ = −1.902, *p* = 0.058, 95% CI [−0.187 to 0.003]). The data are depicted in Fig. 2. The null model had the best fit with a corrected AIC of 3,707.371.

Because of the imbalance of fearful *vs*. neutral trials, we re-ran the analysis including only owls (*n* = 15) that had shown a fearful posture on at least one trial, but the pattern of results was the same.

### Does the familiar human relationship predict the owls’ response?

#### Response

For the model with response as the outcome, the model fit was indicated by a corrected AIC = 752.264 (null model AIC = 732.094, Table S4). Only months (*F*_1, 26_ = 8.198, *p* = 0.008) significantly predicted likelihood of response with months positively associated with likelihood of response (*t* = 2.863, *p* = 0.008, 95% CI [1.004–1.022]), indicating that the longer the caregiver had worked with the owls, the more likely they were to respond to their voice (Table S10).

#### Latency to respond

For the model analyzing effects on latency to respond, the same fixed factors were included in the model. Model fit was indicated by a corrected AIC of 711.250, which was an improvement over the null model (AIC = 728.483, Table S5). There was a significant main effect for months, (*F*_1,__100_ = 9.749, *p* = 0.002), husbandry (*F*_1, 100_ = 7.193, *p* = 0.002) and relationship (*F*_1, 100_ = 5.414, *p* = 0.022). There was a significant positive association between months and latency (*t* = 3.122, *p* < 0.002, 95% CI [0.024–0.106]) such that owls were slower to respond to the familiar voices the longer that caregiver had worked with them. The owls were faster to respond if the keeper engaged in husbandry compared to when they did not (*t* = −7.023, *p* = 0.009, 95% CI [−12.218 to −1.828]). There was a significant negative association between relationship and latency such that the more positive the relationship, the faster the owls responded (*t* = −2.327, *p* = 0.022, 95% CI [−4.530 to −0.360]). See Table S11.

#### Posture

We conducted a GLMM with posture as the outcome using a binomial distribution and logit link function including the same familiar caregiver factors as fixed factors and subject as a random factor. The model fit was indicated by a corrected AIC of 787.634, which was not an improvement over the null model (corrected AIC = 740.346, Table S6). There was a significant main effect of training (*F*_1,__18_ = 9.847, *p* = 0.006) and relationship, (*F*_1,19_ = 5.710, *p* = 0.028). Owls were less likely to show fear if they had been involved in training with the caregiver (*t* = −3.138, *p* = 0.006, 95% CI [0.044–0.539]) and if they were described as having a more positive relationship with that caregiver (*t* = −2.390, *p* = 0.028, 95% CI [0.128–0.875]). See Table S12. Because there were only 11 observations of a fearful posture in 133 trials with familiar caregivers, we re-ran the analysis including only owls (*n* = 15) that had shown a fearful posture on at least one trial, but the pattern of results was the same.

## Discussion

We presented owls in managed care with playbacks of familiar and unfamiliar voices within the same experimental sessions to control for daily variation in extraneous factors, such as temperature, light levels, and ambient noise, and measured whether they made any visible response to the playback based on head turns, vocalizations, locomotion, posture. On only those playbacks that they responded to, we analyzed the speed with which they made a response. We included multiple unfamiliar voice playbacks within the sessions (Saito & Shinozuka, 2013) to allow for disentangling effects of time and responses specific to the fourth, familiar playback. We compared the responses of owls that served as ambassador/program animals with those that were not involved in programs (*i.e*., exhibit owls). Lastly, we examined whether characteristics specific to the owl and the caregiver whose voice was played on the familiar playback predicted their response. For example, we examined months of time spent working with the owl, whether the caregiver engaged in husbandry or training with the owl, and their self-rated relationship quality (higher scores indicated more positive relationships).

The owls were not significantly more or less likely to respond to the familiar playback compared to the unfamiliar playbacks. They did respond more slowly and with increased likelihood of displaying a fearful posture on the familiar playback compared to the first playback. Although this pattern may seem to contradict the notion that faster response latencies are an indicator of vigilance, it is important to note that latency and fearfulness were negatively correlated (ρ = 0.25), which is consistent with this interpretation. The pattern of response latencies was visually consistent with release from habituation to the familiar voice (Saito & Shinozuka, 2013), but the latency to respond to the familiar voice significantly differed only from the latency to respond to the first playback.

Data from the playbacks of familiar voices specifically were of greater interest and provided more compelling evidence that the owls may recognize the voices of their current caregivers. In general, owls were more likely, yet slower, to respond to the voices of caregivers that they had worked with for longer periods of time. Owls also responded more quickly if that caregiver engaged in husbandry, but not training, and if the caregiver had rated the relationship as more positive. This effect is somewhat consistent with findings in domesticated cats where the magnitude of response was stronger for the familiar person’s voice (Saito & Shinozuka, 2013). Importantly, this response has also been shown in non-domesticated cats, such as cheetahs, which responded more quickly and for longer durations to familiar than unfamiliar human voices (Leroux et al., 2018). Crews, Vonk & McGuire (2024) replicated this finding in cheetahs and nine additional species of exotic cats and further demonstrated that cats also responded with greater intensity to familiar compared to unfamiliar human voices. The results were the same when data from the only group-living species—African lions—were excluded. Thus, previous research has shown that non-domesticated and non-group-living species are more likely to respond to the familiar voice. Owls showed a mixed response to the familiar voices. Some factors predicted quicker responses (*i.e*., husbandry and positive relationships) whereas others predicted slower responses (length of the relationship). This may have occurred because the owls’ responses may have sometimes reflected a positive response and sometimes a negative response to the voices. The most common behavioral response we observed involved peering behavior. Although peering can reflect vigilance, it does not necessarily indicate perception of a threat. Slower responses may represent a reduction in vigilance or a decline in attention or interest when a voice becomes familiar. At the same time, owls were more likely to respond at all to caregivers with whom they had longer relationships. Although we were able to document facets specific to the particular relationship between each subject owl and the specific caregivers whose voices were presented on a given trial, it is possible that the vastly different experiences of each owl with humans complicated the pattern of findings.

We did not find overall differences in response, including likelihood of a fearful response between ambassador and exhibit owls. In fact, the owls were less likely to show fear if the caregiver engaged in training with them compared to when they did not. Voices of caregivers who reported having more positive relationships with the owls were also less likely to evoke fear in the owls. It should be noted that observations of fearful posture were relatively rare in the sample—representing less than 10% of the observed trials. This pattern of findings might suggest that training and program experiences do not have adverse effects on these owls.

Because we simply observed whether owls showed a differential response to voices of familiar and unfamiliar humans, and did not require that they make a unique operant response to voices from the two categories, we cannot conclude that these owls categorized familiar voices differently than unfamiliar voices, but their distinctive responses are suggestive that they have this capacity (see also Dutour et al., 2021; Wascher et al., 2012). Prior studies have shown that birds appear to recognize the calls of familiar neighbors of the same species *via* the “neighbor-stranger discrimination” or “dear-enemy phenomenon” in which territorial species respond more aggressively to unfamiliar individuals than toward their neighbors (Temeles, 1994). This phenomenon has been demonstrated in various species of passerine birds (Blumenrath, Dabelsteen & Pedersen, 2007; Brunton et al., 2008) as well as in mammals like the black-and-gold howler monkey (*Alouatta caraya*, Holzmann & Córdoba, 2024) and the European beaver (*Castor fiber*, Rosell & Bjørkøyli, 2002). Of most relevance to the current work, wild tawny owls (*Strix aluco*, Galeotti & Pavan, 1993) and little owls (Hardouin et al., 2007) responded more strongly to unfamiliar than neighbor calls whereas barred owls (*Strix varia*, Odom & Mennill, 2010b) and their closest relatives, the spotted owl (*Strix occidentalis*, Waldo, 2002) did not show significant differences in response to neighbor and stranger duets. It should be noted, however, that Odom & Mennill (2010b) observed trends consistent with the dear-enemy hypothesis and effect sizes comparable to other published studies. Their findings may have been limited by their sample size. A stronger response to unfamiliar voices likely reflects the fact that owl species are generally considered solitary and tend to be territorial, especially when confronted with immediate danger (Odom & Mennill, 2010b).

Although the current results do not provide unequivocal support for the conclusion that owls differentiate the voices of individual caregivers, owls did appear to show reduced responses and fear in the presence of more familiar caregiver voices compared to unfamiliar voices, consistent with prior results from other bird species (Dutour et al., 2021; Wascher et al., 2012). The current study extended previous work to examine the effects of the specific owl/caregiver relationship and found that owls were also quicker to respond to the voices of those that were involved with their husbandry and those that rated their relationships more positively. This is consistent with previous findings that owls that imprint to humans show less distress around them (Potts, 2016), suggesting that they can habituate to human presence (Galeotti & Pavan, 1993).

Even wild birds may enter into symbiotic relationships with humans. Recently, researchers reported that barn swallows showed shorter flight initiation distances to familiar humans, regardless of how much time humans spent in the home where the birds were nesting (Liu, Liu & Liang, 2025). The ability of the swallows to differentiate familiar homeowners from strangers was attributed to their tendency to nest on human structures. Owls are not known to hold such symbiotic relationships with humans, although some species do commonly reside in urban areas where they may come in frequent contact with humans. Urban burrowing owls (*Athene cunicularia*) differ from rural burrowing owls in their flight initiation distance to humans and humans with dogs (Cavalli et al., 2016) and flight initiation distance has been shown to vary in individuals, indicating differences in susceptibility to human disturbance (Carrete & Tella, 2010, 2011). It has been noted that wildlife that adapt to urban environments must demonstrate a degree of tolerance to human presence (Carrete & Tella, 2010, 2013; Samia et al., 2015; Sol, Lapiedra & González-Lagos, 2013) and that this trait may be related to behavioral flexibility and relative brain size (Carrete & Tella, 2011). Owls living under managed care might show greater sensitivity to individual familiar humans compared to wild owls. Behavioral differences in rural and urban populations of burrowing owls may suggest that prolonged exposure to humans acts as a selection pressure on owl behavior and cognition (Carrete & Tella, 2017). However, habituation to human presence appeared to have little impact on individual fear of humans in burrowing owls, which was remarkably consistent over time, suggesting that this individual trait may not be flexibly altered through repeated exposure, at least in this species. Captive birds may show greater responsiveness to familiar human voices because they do not have the option to escape like the wild owls tested by Carrete & Tella (2017).

### Limitations and future directions

Most species are nocturnal and therefore, the owls may have produced stronger responses and more diverse behaviors later into the evening. Some of our owls were pair housed and the behavior of the other owl may have influenced each owl’s own response. Although this introduces an artifact into the study, it also reflects the natural conditions under which the owls are housed. The biggest challenge to interpreting these data and comparing the results to those found with other species is the difficulty in categorizing the observed behavioral response to the stimuli as positive or negative in nature. Given that our subjects likely had vastly different prior and current experiences with humans, it was a challenge to standardize their responses. We considered any head turns, vocalizations, or locomotion as a response to the stimuli (similar to Wascher et al., 2012); however, almost all the observed responses reflected peering type behavior. The same behavior likely indicated different underlying responses for different owls, contributing to some of the noise in our data. We also coded owl posture as neutral, aggressive or fearful. However, there were few instances of the owls appearing fearful and no reliable instances of them appearing aggressive, so we must also consider these results preliminary. We had not initially anticipated that owls would find human voices stress-inducing so we did not plan to collect measures of stress response. Additionally, we did not have any means to collect physiological measures, such as cortisol, from the owls as our study was strictly observational and non-invasive, but we think this would be a valuable addition to future work.

Despite suggestive findings concerning effects of caregiver familiarity, role, and relationship with the owls, these data do not provide unequivocal evidence that owls can recognize the voices of specific individual humans. However, these data add to a sparse literature concerning owl cognition and human/raptor interactions in pointing to various important future directions. Although our overall sample was larger than that used in previous studies with birds (*N* = 14, Dutour et al., 2021; *N* = 8, Wascher et al., 2012), we were limited by a small sample size within each species due to the scarce population of captive owls available for testing. It is possible that owls living in areas with high population density (Madhavan et al., 2025) are more likely to develop the capacity for individual recognition compared to those living in areas with sparser populations. This hypothesis would be of interest for future research; however, the species we tested were not expected to differ in their likelihood to recognize familiar humans. We were primarily interested in how the nature of the caregiver/owl relationship might predict differences in response to caregiver voices and we expected similar results across the owl species. We expected the role of the owl as a program or exhibit animal and the length of the relationship to be a stronger predictor of response than species.

## Conclusion

The existing literature demonstrating recognition of familiar individuals focuses heavily on more social species, whereas less social, pair-bonded species, and territorial species like owls remain severely understudied. Many studies have shown that various species are capable of class recognition; for example, recognizing familiar from unfamiliar conspecifics, but it is more difficult to demonstrate individual recognition (Surányi et al., 2025). Here, characteristics of the specific relationship between the owl and the familiar caregiver predicted aspects of the owls’ responses, which might be indicative of individual recognition. This research contributes to an understanding of how less social species experience day-to-day interactions with humans charged with their care. Understanding how raptors respond to familiar caregivers may have important implications for husbandry decisions and other aspects of care such as their use as program animals. Most of the owls in managed care are exposed to numerous human voices daily whether they take part in programs or not. Our findings suggest that owls in managed care may differentiate the voices of individuals that care for them. This suggests that owls may benefit from some continuity in caregivers. Further, our findings suggest that specific experiences with caregivers, including training, shape owls’ perceptions of those individuals.

## Supplemental Information

10.7717/peerj.21421/supp-1 Supplemental Information 1 Raw data.

10.7717/peerj.21421/supp-2 Supplemental Information 2 Supplemental Tables. Model fits from GLMMs and Parameters from best fitting models.

10.7717/peerj.21421/supp-3 Supplemental Information 3 Codebook for Data.

## Additional Information and Declarations

### Data Availability

The following information was supplied regarding data availability:

The raw data are available in the Supplemental File.
