# Supplementary material for: “Whoooo said that?”: responses of captive owls (Strigiformes) to the voices of familiar caregivers
Source: PeerJ. 2026 Jun 17;14:e21421. doi: 10.7717/peerj.21421 (PMC13282947; doi:10.7717/peerj.21421)
Supplement: Supplemental Information 2 [file peerj-14-21421-s002.docx]

Table S1

| Model | Fixed Effects | Random Effects | K | BIC | AIC | ΔAIC |
| --- | --- | --- | --- | --- | --- | --- |
| **Null** |  | **(1 + Playback / Subject) + (1 /SubjSession)** | **3** | **4317.420** | **4280.660** |  |
| Simple | B + C + BC | (1 + Playback / Subject) + (1 /SubjSession) | 6 | 4381.753 | 4345.097 | 27.677 |
| Full | A (Sex) + B (Status) + C (Playback) + AB + AB + BC + ABC | (1 + Playback / Subject) + (1 /SubjSession) | 11 | 4425.104 | 4388.560 | 107.684 |

Model comparisons for response for all playbacks.

Table S7

Parameter estimates for best fitting model of response for all playbacks

| Predictor | Estimate () | SE | 95% CI | Z | p |
| --- | --- | --- | --- | --- | --- |
| Fixed Effects |  |  |  |  |  |
| Random Effects |  |  |  |  |  |
| **Subject Intercept** | **3.939** | **1.754** | **1.646, 9.426** | **2.246** | **.025** |
| **Playback** | **1.842** | **0.479** | **1.106, 3.067** | **3.844** | **<.001** |
| **Subj/Session** | **4.590** | **0.844** | **3.202, 6.580** | **5.442** | **<.001** |

*Note*. Model Family: Binomial; Link function: Logit. N*_obs_* = 750, N*_sub_* = 21

Table S2

| Model | Fixed Effects | Random Effects | K | BIC | AIC | ΔAIC |
| --- | --- | --- | --- | --- | --- | --- |
| Null |  | (1 + Playback / Subject) + (1 /SubjSession) | 3 | 3742.121 | 3707.969 | 83.57 |
| Simple | B + C + BC | (1 + Playback / Subject) + (1 /SubjSession) | 6 | 3700.985 | 3666.970 | 42.57 |
| **Full** | **A (Sex) + B (Status) + C (Playback) + AB + AB + BC + ABC** | **(1 + Playback / Subject) + (1 /SubjSession)** | **11** | **3658.259** | **3624.400** |  |

Model comparisons for latency for playbacks that evoked a response.

Table S8

Parameter estimates for best fitting model of latency for playbacks that evoked a response

| Predictor | Estimate | SE | 95% CI | t | p |
| --- | --- | --- | --- | --- | --- |
| Fixed Effects |  |  |  |  |  |
| Sex | 1.473 | 3.466 | -5.157,8.459 | 0.476 | .402 |
| Status | .004 | 2.992 | -5.407, 6.348 | 0.157 | .948 |
| **Playback** | **3.911** | **2.580** | **-8.483, 1.656** | **-1.323** | **.004** |
| Sex*Status | .223 | 4.230 | -11.887. 4.734 | -0.845 | .637 |
| Sex*Playback | .396 | 3.431 | -5.585, 7.898 | 0.337 | .811 |
| Status*Playback | .882 | 2.950 | -4.408, 7.184 | 0.471 | .474 |
| Sex*Status*Playback | .729 | 4.178 | -8.183, 8.232 | 0.006 | .572 |
| Random Effects |  |  |  | z |  |
| **Subject Intercept** | **3.939** | **1.754** | **1.646, 9.426** | **2.246** | **.025** |
| **Playback** | **1.842** | **0.479** | **1.106, 3.067** | **3.844** | **<.001** |
| **Subj/Session** | **4.590** | **0.844** | **3.202, 6.580** | **5.442** | **<.001** |

*Note*. Model Family: Linear; Link function: Identity. N*_obs_* = 547, N*_sub_* = 21

Table S3

| Model | Fixed Effects | Random Effects | K | BIC | AIC | ΔAIC |
| --- | --- | --- | --- | --- | --- | --- |
| **Null** |  | **(1 + Playback / Subject)** | **2** | **3738.994** | **3707.371** |  |
| Simple | B + C + BC | (1 + Playback / Subject) | 5 | 3722.134 | 3690.604 | 16.767 |
| Full | A (Sex) + B (Status) + C (Playback) + AB + AB + BC + ABC | (1 + Playback / Subject) | 9 | 4311.637 | 4280.212 | 572.841 |

Model comparisons for posture for all playbacks.

Table S9

Parameter estimates for best fitting model of posture for all playbacks

| Predictor | Estimate () | SE | 95% CI | Z | p |
| --- | --- | --- | --- | --- | --- |
| Fixed Effects |  |  |  |  |  |
| Random Effects |  |  |  |  |  |
| **Subject Intercept** | **1.803** | **0.781** | **0.772, 4.214** | **2.309** | **.021** |
| Playback | 0.297 | 0.210 | 0.074, 1.186 | 1.415 | .157 |

*Note*. Model Family: Binomial; Link function: Logit. N*_obs_* = 750, N*_sub_* = 21

Table S4

| Model | Fixed Effects | Random Effects | K | BIC | AIC | ΔAIC |
| --- | --- | --- | --- | --- | --- | --- |
| **Null** |  | **(/ Subject) + (1 /SubjSession)** | **1** | **740.940** | **732.094** | **20.17** |
| Full | Months + Husbandry + Train + Relationship | (/Subject) | 5 | 761.024 | 752.264 |  |

Model comparisons for response on familiar playbacks only.

Table S10

Parameter estimates for best fitting model of response on familiar playbacks only.

| Predictor | Estimate | SE | 95% CI | t | p |
| --- | --- | --- | --- | --- | --- |
| Fixed Effects |  |  |  |  |  |
| Random Effects |  |  |  | z |  |
| **Subject Intercept** |  |  |  |  |  |
| **Subj/Session** | **3.004** | **1.362** | **1.236, 7.304** | **2.206** | **.027** |

*Note*. Model Family: Binomial; Link function: Logit. N*_obs_* = 150, N*_sub_* = 21

Table S5

| Model | Fixed Effects | Random Effects | K | BIC | AIC | ΔAIC |
| --- | --- | --- | --- | --- | --- | --- |
| Null |  | (/ Subject) + (1 /SubjSession) | 2 | 733.653 | 728.483 | 17.233 |
| **Full** | **Months + Husbandry + Train + Relationship** | **(/Subject) + (1 /SubjSession)** | **6** | **716.337** | **711.250** |  |

Model comparisons for latency for playbacks that evoked a response on familiar playbacks only.

Table S11

Parameter estimates for best fitting model of latency for playbacks that evoked a response on familiar playbacks only.

| Predictor | Estimate | SE | 95% CI | t | p |
| --- | --- | --- | --- | --- | --- |
| Fixed Effects |  |  |  |  |  |
| **Months** | **9.749** | **0.021** | **0.024, 0.106** | **3.122** | **.002** |
| **Husbandry** | **7.193** | **2.619** | **-12.218, -1.828** | **-2.682** | **.009** |
| Training | 2.146 | 2.376 | -8.195, 1.233 | -1.465 | .146 |
| **Relationship** | **5.414** | **1.051** | **-4.530, -0.360** | **-2.327** | **.022** |
| Random Effects |  |  |  | z |  |
| **Subject Intercept** | **49.266** | **8.048** | **35.768, 67.857** | **6.121** | **<.001** |
| Subj/Session | 5.577 | 7.157 | 0.451, 69.002 | 0.779 | .436 |

*Note*. Model Family: Linear; Link function: Identity. N*_obs_* = 105, N*_sub_* = 21

Table S6

| Model | Fixed Effects | Random Effects | K | BIC | AIC | ΔAIC |
| --- | --- | --- | --- | --- | --- | --- |
| **Null** |  | **(/ Subject) + (1 /SubjSession)** | **1** | **748.949** | **740.346** |  |
| Full | Months + Husbandry + Train + Relationship | (/Subject) | 5 | 796.143 | 787.634 | 47.288 |

Model comparisons for posture on familiar playbacks only.

Table S12

Parameter estimates for best fitting model of posture on familiar playbacks only.

| Predictor | Estimate | SE | 95% CI | t | p |
| --- | --- | --- | --- | --- | --- |
| Fixed Effects |  |  |  |  |  |
| Random Effects |  |  |  | z |  |
| **Subject Intercept** | **2.655** | **1.306** | **1.012, 6.965** | **2.032** | **.042** |

*Note*. Model Family: Binomial; Link function: Logit. N*_obs_* = 150, N*_sub_* = 21
