# Supplementary material for: “Whoooo said that?”: responses of captive owls (Strigiformes) to the voices of familiar caregivers
Source: PeerJ. 2026 Jun 17;14:e21421. doi: 10.7717/peerj.21421 (PMC13282947; doi:10.7717/peerj.21421)
Supplement: Supplemental Information 3 [file peerj-14-21421-s003.docx]

Owl Codebook

Sex : 0 = female, 1 = male

Status: 0 = exhibit, 1 = ambassador

Husbandry: 0 = no, 1 = yes

Training: 0 = no, 1 = yes

BehCat: 0 = no response, 1 = response

Voice: 0 = unfamiliar, 1 = familiar

Posture: 0 = neutral, 1 = fearful
